# How Do Nigerian Newspapers Report Corruption in the Health System?

**DOI:** 10.34172/ijhpm.2020.37

**Published:** 2020-03-14

**Authors:** Mohammed Abba-Aji, Dina Balabanova, Eleanor Hutchinson, Martin McKee

**Affiliations:** Faculty of Public Health and Policy, London School of Hygiene & Tropical Medicine, London, UK.

**Keywords:** Corruption, Media, Governance, Nigeria

## Abstract

**Background:** Nigeria has a huge burden of corruption, with the health system especially vulnerable. The media can play a role in tackling it, by shaping the narrative around it. However, its influence depends on the extent and framing of its reporting on corruption. This paper reviews, for the first time, coverage of corruption in the health system in the Nigerian print media.

**Methods:** The top 10, by circulation, newspapers in Nigeria were selected and searched using the LexisNexis database for articles covering corruption in the health sector over a 2-year period (2016–2018). Two newspapers are not included in the database and were searched manually. 135 articles were identified and subject to content and framing analyses.

**Results:** The Punch newspaper had the highest number of publications focussed on corruption in the health sector. The National Health Insurance Scheme (NHIS) was the organization attracting most coverage, followed by the Federal Ministry of Health. Corruption in the health sector was predominantly framed as a political issue. Most coverage was episodic, focused on the details of the particular case, with much less thematic, delving into underlying causes. Corruption was most often attributed to a lack of accountability while enforcement was the most frequent solution proffered.

**Conclusion:** This study highlights the potential role of media analyses in helping to understand how newspapers cover corruption in the health sector in Nigeria. It argues that the media has the potential to act as an agent of change for tackling corruption within the health sector.

## Introduction

A recent paper in this journal called for a more open discussion of corruption in the health sector, noting how there is often a reluctance to confront it even though there is widespread, but unspoken, acceptance of the pernicious effects of corrupt behaviour.^1^ Yet as Mackey notes, the situation is changing, with politicians more willing to challenge practices that were long taken for granted.^2^ Politicians do not act in a vacuum and their actions are frequently influenced by popular opinion, which in turn is influenced by the mass media. Thus, there is some empirical evidence linking media freedom with lower levels of corruption.^3^ Researchers have examined how corruption is represented in the media but apart for some research in South Africa, have paid very little attention to the health sector.^4,5^ In this paper we report how newspapers in Nigeria, addressed health sector corruption, asking how it is framed, who is held responsible, and what solutions are proposed.

Our study is set in Nigeria, a country facing a major challenge from corruption, defined by Transparency International as the “misuse of public trust for private gains.”^6^ Although official figures are lacking, in 2006 Nuhu Ribadu, chair of the Nigerian Economic and Financial Crimes Commission claimed that “Nigeria has lost over $384 billion to corruption since its independence in 1960.” A 2017 survey undertaken by the United Nations Office on Drugs and Crime, working with the Nigerian Bureau of Statistics estimated that about “400 billion Nigerian Naira, the equivalent of $4.6 billion in purchasing power parity” was paid as bribes within the previous year.^7^ Transparency International’s 2018 corruption perception index ranked Nigeria 144 out of 180 countries surveyed (where 1, Denmark, is perceived as the least corrupt).^8^ The health sector in Nigeria is considered especially vulnerable and a recent systematic review identified 5 forms that it takes in health systems in Anglophone West Africa: absenteeism; diversion of patients to private facilities; inappropriate procurement; informal payments; and theft of drugs and supplies.^9^

The role that the media can play in tackling corruption in both high- and low-income settings has long been recognised, with recent research identifying campaigning newspapers as important partners for coalitions seeking to act against corruption in low- and middle-income countries (https://ace.soas.ac.uk/486-2/).

This role has been endorsed by a number of international agencies, such as the Organisation for Economic Co-operation and Development, which states that “The media and investigative journalism play a crucial role in bringing allegations of corruption to light and fighting against impunity” in a report that provides a detailed review of the contribution that investigative journalism has made to the fight against corruption, including a series of case studies.^10^ The Council of Europe has produced guidance for journalists reporting corruption.^11^ This provides a useful review of the ethics of journalism in reporting on this topic, most recently set out in the 1971 Munich Declaration on Duties and Rights of Journalists. The Council of Europe guidance covers such issues as when it is appropriate to publish the identity of an individual who has not been convicted of an offence, standards of evidence, when it is justified to investigate corrupt behaviour, protection of journalists and their sources, and the need to avoid what is termed “tabloid lynching.”

Coverage of corruption by the media may have tangible and intangible effects.^12^ The former includes explicit mentions of corruption, such as the initiation of a formal investigation and removal of corrupt politicians and officials involved in fraudulent activities. Intangible effects include where the media promotes broader changes in governance such as accountability of public officials, politicians and institutions. Beyond the importance of corruption, Nigeria is an appropriate setting for such a study as it has one of the most vibrant and varied media landscapes in Africa. It has over 100 national and local publications, many with an online presence, and a willingness to criticize government policies and politicians openly. This can be seen in a study by Fadairo et al who undertook a content analysis of coverage of corruption in major Nigerian newspapers. They found that coverage had increased between 2006-2010.^13^ However, most articles were short and on inside pages, which they interpreted as indicating the low priority given to corruption-related news. Most stories examined politics and governance, with least attention given to agriculture, transport, and the energy sector. The health sector did not feature in their data but their study was undertaken before the recent increase in attention it has attracted from the Nigerian government and the international community.

While that study showed that newspapers are willing to report corruption, it is also important to be aware that narratives of corruption in the media can be part of corrupt practices themselves in an activity locally known as so-called “brown envelope journalism,” whereby journalists are paid to write stories favourable to individuals or organisations.^14^ Moreover, the media advocacy group, Freedom Media House, describes the press in Nigeria as only partly free.^15^ While the government passed a freedom of information act in 2010,^16^ journalists still risk prosecution for contravening a broadly termed “Cyber Security Crime Act” and while no prosecutions have taken place, journalists face a range of other threats.^15^

Our study is timely. The study by Fadairo et al is now almost a decade old and, in the intervening period, health systems corruption has risen high on the national and international agenda, being seen as an important barrier to achieving the Sustainable Development Goals.^17^

## Methods

### Theoretical Approach

Our approach to communication uses framing theory, which considers the social construction of a social phenomenon.^18-20^ Framing theory has been useful in making sense of global health governance,^18^ and has been used to make sense of successful discourses of tobacco control and anti-microbial resistance. It comprises a set of concepts and theoretical perspectives on how individuals, groups, and societies go about organising, perceiving and communicating about reality. To frame, according to Entman is to “select some aspects of a perceived reality and make them more salient in a communicating text in such a way as to promote a particular problem definition, causal interpretation, moral evaluation, and treatment recommendation for the item described.”^21^ Any of these 4 framing functions can be found in a communicated message although many sentences in a text may perform none of them. Salience, according to Entman, means “making a piece of information more noticeable, meaningful, or memorable to the audience.” The more salient an issue is, the more likely it will be remembered by the audience. A frame could direct public attention towards or away from an aspect of perceived reality. Framing theory has been used to study (separately) both health and corruption-related issues in Nigerian newspapers.^22,23^ However, it has not, to our knowledge, been used to study newspaper framing of corruption specifically within the health sector in Nigeria or elsewhere.

Framing as described by Iyengar categorises news stories into *episodic and thematic* frames.^24^ Episodic frames depict issues involving specific instances that do not consider the broader perspective or context within which these issues occur. An episodic framing will thus obfuscate the causes, with the narrow understanding often leading to simplistic or counterproductive solutions, such as repeated calls for enforcement and punishment without considering why these measures, on their own, are ineffective. Thematic frames, on the other hand, depict issues more broadly, placing them in a relevant socio-political context. This type of framing is complex and requires a degree of knowledge and an understanding of nuances in the country’s political and health systems.

## Aim

The aim of this study is to assess how corruption within the health sector was framed in Nigerian newspapers between July 2016 and July 2018. It asks 5 questions:

1. How do newspapers define the problem of corruption in the health sector?
2. What do newspapers report as the causes of corruption in the health sector?
3. What do newspapers report as solutions for tackling corruption within the health sector?
4. How do Nigerian newspapers frame health sector corruption?
5. What solutions, involving which actors, can play a role in tackling corruption in the health sector?

### Selection of Articles

We initially used the LexisNexis database, the most extensive database of newspaper stories. It is updated weekly and includes almost all major Nigerian newspapers. Our search used the following filters: country-Nigeria, type of publication-Daily publication; publication period-July 2016-July 2018; language-English (there is no option for local Nigerian languages on the database); word limit-no filter for min/max selected.

We cross-referenced these results with data from the Advertisers Association of Nigeria (ADVAN) to obtain circulation figures. Two national daily newspapers, The Punch and Guardian, have the highest circulation but are not included in the LexisNexis database. Hence, these were included along with the top 8 of the national dailies on the ADVAN list. All the newspapers selected were national dailies. The newspapers thus selected were the 10 most widely read, with the broadest coverage in Nigeria’s 6 geopolitical zones.

The newspapers included were then searched, using LexisNexis for the 8 within it, with the other 2 being searched, in their online editions, manually. We sought to identify articles, defined as news reports, editorials, opinions, interviews, letters and commentaries. The search strategy was designed to identify news stories relevant to corruption in the health sector, published between July 2016 and July 2018, in English (which is widely spoken in Nigeria). The strategy sought articles that included health-related terms (eg, health OR medical OR hospital) AND those related to corruption (eg, Corrupt* OR Embezzle* OR Fraud* OR Misappropriat* OR Bribe* OR Informal payments OR Scam). In both cases we added variants and synonyms to maximise the yield such as “anti-corruption” “anticorruption” and “anti corruption.”

The search in LexisNexis database yielded over 3000 hits. After removing duplicates, this was reduced to 2983. These were reviewed individually to exclude those that were not relevant to corruption in the health sector. The exclusion criteria were;

1. News stories focusing on the president’s health and its impact on tackling corruption;
2. News stories metaphorically referring to corruption as a disease affecting the health of the nation and
3. News stories about the health of persons standing trial for corruption eg, person accused of corruption being granted bail due to ill health.

A similar approach was used to retrieve newspaper articles from The Punch and Guardian. This left 113 articles from LexisNexis and 58 articles from The Punch and Guardian Newspapers.

The next stage involved screening to exclude unspecific reports such as (1) “Health minister urges health workers to shun corrupt practices” and “President promising to fight corruption in all sectors.” We also excluded articles that covered corruption in other sectors that contribute to population health, such as food safety. This is a large area that would require a separate study. The final total number included for framing and content analysis was 135. The process is summarised in a PRISMA chart in Supplementary file 1.

### Analysis of Articles

We used the directed approach to content analysis described by Hsieh and Shannon.^25^ They define qualitative content analysis as “a research method for the subjective interpretation of the content of text data through the systematic classification process of coding and identifying themes or patterns.” As this method adopts an iterative approach, codes were created deductively using existing literature and inductively from an initial coding of 5 randomly selected articles by MA and DB. This approach is recommended by framing scholars such as de Vreese^26^ and Entman.^21^ After assessing intercoder reliability, the 135 news stories were coded by MA. This involved a 4-stage strategy. These stages were: coding variables for tone, publication source, date, and word count (stage 1); type of corruption as categorised by Transparency International (stage 2); content analysis with coding by framing functions (cause, impact, stakeholder responsible or tackling corruption, means to tackle it) (stage 3); and framing analysis (episodic or thematic frames) (stage 4). These are described in more detail in Supplementary file 2.

Articles were coded under thematic frames if they considered other systemic explanations about the causes, impacts and solutions for corruption in the health sector. Two thematic frames emerged from close reading of the first 5 articles. These were *bureaucratic or political* framing of corruption. The criteria for coding articles as political or bureaucratic include (1) identification of the role of politics or bureaucracy as a cause of corruption in the health sector, and (2) recommending action for bureaucrats or politicians to pursue in order to tackle corruption. Similarly, criteria for coding articles as using individual or organizational frames include (1) blaming individuals or organizations for corruption in the health sector and (2) calling on individuals or organisations to address the issue of corruption in the health sector. For news stories that covered both frames, the frame with the more extensive coverage as indicated by higher word count was coded.

## Results

The Punch newspaper, with 27, had the highest number of references to corruption in the health sector. The Sun and Daily Trust came next, with 22 and 20, respectively. Most articles were news stories (67%), followed by editorials (15%) and opinions (11%). Features, commentaries and interviews made up less than 10% of all the articles analysed. Further details are in Table 1. The average story length was 542 words, while the longest and shortest were 2247 and 167 words, respectively.

**Table 1:** Characteristics of Articles Analysed

**Newspaper**	**Circulation Figures**	**Geographical** **Spread**	**Articles Analysed**							
			**News Stories**	**Editorial**	**Op-Ed**	**Feature Article**	**Interview**	**Commentary**	**Total**	**Percentages of Newspaper Publications by Company**
Daily Independent	2000	Nationwide	9	3	0	0	0	0	12	8.89
Daily Trust	11 672	Nationwide, Dominant in North	16	0	0	4	0	0	20	14.81
Guardian	25 222	Nationwide	4	0	2	1	0	0	7	5.19
National Mirror	2000	Nationwide, South West	2	0	0	0	0	0	2	1.48
Nigerian Tribune	8314	Nationwide	15	0	1	0	0	0	16	11.85
Punch	34 264	Nationwide	20	2	2	2	0	1	27	20.00
The Nation	28 000	Nationwide	7	7	0	1	0	0	15	11.11
The Sun	25 632	Nationwide	4	6	10	1	1	0	22	16.30
Thisday	21 703	Nationwide	7	1	0	0	0	0	8	5.93
Vanguard	25 241	Nationwide	5	1	0	0	0	0	6	4.44
Total	184 048		89	20	15	9	1	1	135	100.00
		Percentages of types newspaper articles	65.93	14.81	11.11	6.67	0.74	0.74	100	

### Content Analysis

The results of the content analysis are summarised in Table 2. The most frequent framing function addressed was *means to tackle corruption* with 50 references (33.6%), followed by *impact of corruption on health* with 29 references. Fewer articles examined *responsibility for tackling corruption* or *causes of corruption.* We now turn to each of these.

**Table 2:** Content Analysis of Framing Functions

**Framing Functions**	**N = 149 (100%)**
**1. Causes of corruption**	n = 18 (12%)
a. Lack of accountability.	10 (6%)
b. Discretionary power amongst officials	4 (2%)
c. Lack of transparency	2 (1%)
d. Absence of citizen’s voices	2 (1%)
e. Monopoly of government provision	0 (0%)
**2. Impact of corruption**	n = 29 (19.4%)
a. Systemic impacts	6 (4.0%)
b. Health system impacts	20 (13.4%)
c. Health outcome impacts	3 (2.0%)
**3. Responsibility for tackling corruption**	n = 22 (14.8%)
a. Regulator	6 (4.0%)
b. Provider	0 (0%)
c. Patients	2 (1.3%)
d. Politicians	14 (9.4%)
**4. Means to tackle corruption**	n = 50 (33.6%)
**a** ***. Reducing opportunities for corruption***	n 1 = 26 (17.4%)
i. Detection and enforcement	10 (6.7%)
ii. Improving accountability	7 (4.7%)
iii. Promoting citizens’ voices	3.(2.0%)
iv. Reducing discretionary powers	3 (2.0%)
v. Reducing monopoly of providers	3 (2.0%)
***b. Reducing pressures for corruption***	n 2 = 5 (3.3%)
i. Increasing health workers wages	2 (1.3%)
ii. Extending universal health coverage	3 (2.0%)
***c. NHIS specific solutions***	n 3 = 19 (12.8%)
i. Decentralizing the scheme	1 (0.7%)
ii. Improving payment methods	1 (0.7%)
iii. Improving information systems	3 (2.0%)
iv. Scrapping HMOs	8 (5.4%)
v. Remittance of embezzled funds	6 (4.0)

Abbreviations: HMOs, health management organizations; NHIS, National Health Insurance Scheme.

Causes of corruption was the framing element least frequently covered, in only 18 articles. Of interest is the way in which the causes reflect the good governance model of corruption with its concerns with accountability, discretion, transparency and citizen voice.^27-29^ The cause most commonly reported was *Lack of accountability* due to lack of enforcement (10 references), followed by *excessive discretionary powers* (4 references) of health sector officials, and then *lack of transparency* and* absence of citizens’ voices* with 2 references each. No article cited *monopoly of government provision* as a cause.

Impacts of corruption were categorised into systemic(societal) impacts, impacts on the health system, and on health outcomes. Systemic impacts are on society more generally, for example when a family pays informally the money spent is not available for education. Health systems impacts are those that directly affect the health system, such as loss of funds to a health facility due to diversion or embezzlement of funds. Health outcome impacts are those that directly impact on an individual’s health, such as death due to counterfeit drugs or absenteeism by health workers. Among them, the impact on the health system was most frequently included (20 references), heavily outweighing systemic (6 references) and health outcome impacts (3 references). The most reported health system impact was inequality of access to health services (8 references), followed by reduced funds for the health sector (5 references).

Means to tackle corruption was the element that featured most in the newspapers studied. For the most part, the articles present standard interventions drawn from ‘good governance’ models created at the World Bank in the 2000s that have been adapted for the health sector and include reducing opportunities for corruption, with better detection, improved systems of accountability, more opportunities for citizens to have a voice, reduction in the discretionary powers of officials, and eliminating the provider monopoly in the public sector.^27-29^ A second set of proposals advocated measures to reduce pressures for corruption, including better salaries and expansion of coverage. There were a few articles (discussed in more detail later) that dealt with specific aspects of the National Health Insurance Scheme (NHIS), mostly linked to a crisis in the relationship between the NHIS and the health management organizations (HMOs) that now provide care. Finally, one article advocated decentralizing the NHIS to create health insurance schemes within each state.

Few articles allocated responsibility for corruption, but of those that did, most held politicians accountable. An example is the confusion that arose when the NHIS Executive secretary refused to be fired by the minister of health, a decision later reversed by the president to retained him in post.

We now turn to the types of health sector corruption reported. The most frequent were financial and workforce management, with 27 references. Within them, embezzlement and misuse of national funds were most often reported, in 17 articles. This was followed by embezzlement and misuse of donor funds, in 7 articles. The second most frequently reported type of corruption was procurement, including ineffective procurement by government, often referred to as “White Elephant Projects.” Even though many reports of corruption fell within the remit of the health sector, specifically the NHIS, articles mainly focused on financial and workforce mismanagement within the organisation itself and only occasionally on health system regulation. Finally, a considerable volume of coverage related to one incident, termed NHIS-gate, which had many characteristics that encouraged sensationalist reporting (Box 1).

**Box 1. NHIS-GATE**

HMOs had developed initially as entities providing private health insurance but, with the creation of the NHIS, some of them assumed responsibility for distributing its funds, in parallel with their private business.^30^ In 2017 some were accused of large scale embezzlement and mismanagement of substantial national funds, in collusion with the former executive secretary of the NHIS.

The first allegation of corruption was directed at the previous administration of the NHIS, which was accused of colluding with HMOs to embezzle national funds to the tune of $2.8 million (N1.05 billion).

The incoming executive secretary played a prominent role in drawing this to public attention, blowing the whistle at a parliamentary public hearing. This added to the drama. The media reported the story with captivating headlines such as “NHIS-SAGA” and “NHIS-GATE.” However, in a twist of events, the new executive secretary, Usman Yusuf, was himself accused of corruption and was suspended by the Minister of Health. Yusuf refused to comply with this directive, saying that only the president, who at the time was outside the country, could sack him. This prolonged dispute gained much media attention. The solution most often proposed, summarised as “Scrapping the HMOs” reflected widespread discontent with them, captured by Yusuf’s description of them as “Bloodsucking leeches” responsible for the “rot in the system.” Other solutions proposed were the recovery of the embezzled funds from the accused HMOs and the individuals involved.

Abbreviations: HMOs, health management organizations; NHIS, National Health Interview Survey.

### Framing Analysis

Corruption within the health system was often framed as a political issue that thrives because of the lack of political will to tackle corruption more generally. This was attributed, in several articles, to the existence of a 2-tier system, whereby the political elite do not use public health services but instead travel abroad, often to high income countries, to receive care, so are indifferent to the quality of services in Nigeria. Examples include:

*“That is, now that your life has been saved in faraway London by the way of our common wealth, where art thou about the lives of other Nigerians?...Mr. President, it seems like only yesterday when you raised our hopes by campaigning against medical tourism that not only drains the national treasury but is also the sole province of the rich and famous”* (The Sun, October 9, 2017).

*“The public healthcare system in the country is going through some of the worst forms of criminal negligence and abandonment. One reason for this is the phenomenon of foreign medical tourism by most government officials and their family members”* (The Sun, October 12, 2017).

*“It is indeed shameful that the health sector has been left to rot away while the political class indulges in medical tourism which serves as a means of coveting monetary gains in Estacodes [a term used in former British colonies to describe travel allowances]”* (Daily Independent, October 18, 2017).

More specific, episodic frames dominated the news reports, in 71% of articles, with thematic frames adopted in the remaining 29%. Thus, Nigerian newspaper readers would have little exposure to an in-depth discussion of underlying causes. Eighty percent of the thematic frames were in the editorial and opinion section of 1 newspaper, The Sun.

When thematic framing was used, it represented corruption in the health sector as either political or bureaucratic, with the former dominating, seen in 35 articles while only 7 framed it as a bureaucratic issue.

Political framing tended to focus on political affiliations and roles played by politicians in instances of corruption in the health sector. Political framing was most common in The Punch newspaper, which has the highest readership in the country. Interestingly, even when a case of corruption clearly involved actions by bureaucrats or failure to apply official procedures, such as internal audits, the criticism was often shared with politicians and solutions proposed were often political. An example of malfeasance committed by an official where this happened included:

*“Extant financial regulations at the agency (NHIS) stipulates that monetary approvals in excess of N2500000 must be ratified by the board before approval for disbursement must be given. However, a former Executive Secretary reportedly unilaterally paid N1.05 billion to some HMOs under the guise of arrears without approval or authorisation from the board”* (Nigerian Tribune, June 11, 2017).

These articles frequently criticised the president for maintaining double standards in the fight against corruption. Others, however, praised him for fighting corruption while urging him to do more. This in some instances could be attributed to the president’s very public stance against corruption. For example, the reinstatement of the suspended executive secretary of the NHIS was framed as political even though many viewed it as an issue of governance. The following quote from Daily Independent newspaper illustrates this framing:

*“According to him, the presidential directive reinstating Mr. Yusuf without trying him, is no doubt, a continuation of the body language that some people have immunity against trial for allegations of corruption, adding this is nothing but an unacceptable addition to indices of political impunity under the APC [All Progressives Congress, the President’s party] regime”* (Daily Independent, February 10, 2018).

Framing of health sector corruption as either political or bureaucratic included discussion of causes, solutions, and attribution of responsibility. Examples are summarised in Tables 3 and 4.

**Table 3:** Analysis of Political Thematic Framing of Corruption in 35 Articles

**Framing Functions**	**Illustrative Examples**
Causes	The Sun October 13, 2017 The damning report on Aso Rock Clinic (1) *“The public healthcare system in the country is going through some of the worst forms of criminal negligence and abandonment. One reason for this is the phenomenon of foreign medical tourism by most government officials and their family members.”* *“Issues of medical tourism and the different levels of corruption that have led to the collapse of public healthcare. The shame of the entire drama is that the political elite have sinned and fallen short of the glory of exonerating themselves from these vices tearing down our public healthcare.”*
Impact	Punch January 14, 2018 Patients dying from lack of oxygen in Nigerian hospitals *“What can one say in a situation in which patients die for a lack of medical oxygen? It was once my misfortune to observe the serial death of some four patients in an intensive care unit….The bottom line is of course, corruption*.” Punch February 26, 2017 How corruption affects healthcare *“This is because when you have healthy citizens, they are more productive, enterprising and much less likely to engage in activities which might endanger their health or their lives while in active pursuit of their daily bread. They will not indulge in prostitution and certain kinds of criminal activity or use narcotics.”* *“What we have been witnesses to is a systematic degradation of quality hospital care that is vastly at odds with the 21st Century. We now see hospitals which in the recent past were able to conduct total knee replacement and open heart surgery gradually becoming unable to do so.”* *“Very often, between the various agencies and the hospitals where the funds are destined to be spent, a significant percentage finds its way into the pockets of powerful people. As a result, the village wallows in poverty and disease, while the chief of the clan enjoys the spoils of his high office.”*
Responsibility for addressing corruption	The Nation October 13, 2017 Much ado over Ibom Hospital's closure *“Uyoatta added: 'The Ibom Specialist Hospital was built by Senator Akpabio to massage his political ego, especially for him to be seen as having achieved above his predecessors.”* *“I am of the strong belief that the project was used as a 'drain hole' to funnel the state's oil resources into private pockets, and the result is what we are seeing today with the closure of the hospital which did not even have any economic benefits to the state.”*
Solutions proposed	The Nation September 5, 2017 Doctors Strike: Mixed reactions in Asaba, Ilorin *“'Corruption is no more a big word in Nigeria, we appeal to the Senate to establish a committee to probe the teaching hospitals and see what is going on.”*

**Table 4:** Analysis of Bureaucratic Thematic Framing of Corruption in 35 Articles

**Framing Functions**	**Illustrative Examples**
Causes	The Sun October 31, 2016 For a viable Health Insurance Scheme *“Not helping matters in the sad fate of SHIPs in Nigeria is the inability or refusal of NHIS to stamp its regulatory and enforcement authority on HMOs and HCPs.”*
Impact	Guardian May 9, 2017 Fraud allegations threaten universal health scheme *“Ewenla said the implication of this alleged distortion was that the healthcare providers would not have the fund to provide services as earlier designed, resulting in the enrollees being denied access by facilities.”* *“Sanda noted that the development had resulted in the re-introduction of out-of-pocket payment, which the scheme was designed to completely eliminate.”*
Responsibility for addressing corruption	The Sun October 4, 2017 Nigeria's poor healthcare governance *“Above all, the minister of health must show strong resolve in ensuring efficient healthcare governance and management of their workforce. Any nation that wants to boast of a viable economy must have a vibrant health sector.”*
Solutions proposed	The Sun October 13, 2017 The damning report on Aso Rock Clinic (2) *“The National Health Insurance Scheme must be reformed to efficiently provide services to poor Nigerian and get millions of Nigerians to enrol. Besides, the corruption that surfaced recently at the NHIS must be frontally tackled and the dual principles of transparency and accountability restored.”*

Abbreviations: HMOs, health management organizations; NHIS, National Health Interview Survey; HCPs, Healthcare providers; SHIPs, Social Health Insurance Schemes.

The tone of the reporting was largely negative, seen in 100 (74% of the articles), while the remainder took a neutral stance.

## Discussion

We analysed 135 articles on corruption in the health sector published in leading Nigerian newspapers between July 2016 and June 2018. The tone of reporting was mostly negative across all the reports analysed. The framing element used most was the need for solutions to corruption, followed by responsibility for tackling it. Causes of corruption featured least. The type of corruption most often reported was Financial and workforce management followed by Procurement. Most attention focused on the role of the NHIS. Episodic framing dominated, describing what happened but not placing it in a broader context. Thematic framing, used in a minority of articles, focused on political rather than bureaucratic issues.

Episodic framing focuses the blame on individual perpetrators, portraying actors as criminals or heroes. As such, it encourages simplistic solutions, such as those based on enforcement and detection. This does find support from economic theory, which sees public officials engaging in corrupt practices if the benefit of doing so exceeds the cost.^31^ However, while clearly part of any solution, it ignores the factors that create the incentives to act corruptly and its dependence on effective institutions. In Nigeria, there is a risk that, without attention to these considerations, investment in a system of enforcement, possibly involving new cadres of inspectors and enforcers, could simply increase the scope for corrupt practice. This is an important concern given evidence implicating the police and judiciary in corruption allegations.

The existence of newspaper reports focusing on the fundamental causes of corruption in the health sector, such as *“Corruption and the health sector”* (The Nation (Nigeria), October 27, 2017) shows that the media can critically appraise this issue. Reports such as this can help place corruption higher on the national agenda by framing it as a political issue and calling on the public and politicians alike to take action. However thematic framing is challenging for a journalist without the requisite knowledge of the workings of the health system. It requires an understanding of the inherent vulnerabilities that promote corruption within the health system such as asymmetry of information, the inevitable clinical uncertainty, and power imbalances, all affecting transactions that take place largely away from public view.^31^

There is growing understanding in Africa on the importance of investigative journalism in tackling corruption.^32^ A study based on interviews with Nigerian journalists^33^ demonstrated the journalists were familiar with the principles of investigative journalism but they felt that it was poorly developed in the country. Barriers to doing so included clientelism, poor remuneration, bad working conditions, and corruption within the media, including the relationship between publishers and politicians. This includes the “brown envelope” journalism referred to above, involving the transfer of resources between sources and journalists.^34^ However, there are signs that this is no longer being tolerated, with the Nigerian anti-graft agency recently seizing the premises of The Sun newspaper, citing concerns about the origin of the funds used to build the company.

There is also growing evidence that media coverage of corruption can be effective, although inevitably it is difficult to establish a precise cause and effect relationship. Thus, the Nigerian parliament recently passed legislation banning state sponsored medical tourism, an issue which, as noted above, had featured in media stories. In another case, BBC reporters went underground to investigate the epidemic of drug abuse in Nigeria. The investigators were able to catch on camera how a major drug company was producing large quantities of Codeine cough syrup and selling them off on the black markets to drive profit, further fuelling the addiction epidemic. The government responded the day after the documentary was aired, by ordering closure of the plant and dismissal of some of the pharmaceutical representatives involved.^35^

While thematic framing can highlight fundamental causes, it is not without its drawbacks. Thematic framing can have the unintended consequence of undermining the citizen’s confidence in their ability to contribute personally to tackling corruption in the health sector. This reductionist view can further perpetuate corruption if the public feels that whatever they do amounts to nothing.

We found that thematic framing often pointed to the role of the political elite. However, this also tended to focus on improved enforcement and detection, without considering the underlying reasons why corruption persists. In particular, there was little attention paid to the power structures that underpin corrupt behaviours, or how they might be challenged by, for example, strengthening the voice of health workers seeking to deliver high quality care. Finally, the emphasis on the politics of corruption in the media suggests that discourses of corruption (including within the health sector) may be connected to political competition more generally and perhaps less concerned with stamping out corruption per se. This has been observed in Argentina and Chile,^36^ where it was shown that scandals often became public not because of a desire to tackle corruption but rather because those individuals leaking information were seeking to damage others, often within their own parties, and thus gain power.

This study has strengths, but also some limitations. It is the only research, to our knowledge, that has used LexisNexis Academic, the most comprehensive database of newspaper articles, to examine newspaper reports of corruption in the health sector. However, 2 of the leading newspapers in Nigeria are not included so it was necessary to search them manually. The search was highly structured and involved a multi-stage process to identify relevant articles. However, constraints on resources meant that it only covered a 2-year time period. This does cover a time when corruption in the health sector has been rising higher on the agenda and is of clear contemporary importance, but it would be interesting to see if the nature of reporting had changed over time. Second, we only included English language newspaper, and while the national print media is overwhelmingly in English,^37^ it is possible that coverage may differ in some local non-English newspapers. Third, Nigeria is a federal country, with important political, religious, and cultural divisions. A previous study did find some regional differences in coverage of 2 major scandals^38^ but it was not possible to look for this with the limited number of articles on any one topic. Fourth, the selection of newspapers was based on a listing compiled by the ADVAN. The ranking has been contested by 2 newspaper companies but no alternative exists. It was also confined to the 10 largest circulation papers, due to logistic constraints, while there are over 67 daily newspapers in Nigeria. However, those included have over 90% of the overall market share. Finally, the study only included print media, while television and radio are also important sources and electronic media are becoming more widely used. However, there is no simple way of searching these sources systematically.

As noted above, this is a relatively small scale study, undertaken with limited resources. Future research should extend the time period studied, providing a historical perspective, even, perhaps, extending to before the re-emergence of democracy in 1999. It would also be useful to understand better the knowledge, attitudes, and practices of Nigerian journalists confronted by evidence on corruption in the health sector. Looking beyond newspapers, it will also be important to examine other media outlets, including television and radio, but also online sources. The last of these will become even more important given the scope for manipulation with what is termed “fake news” or disinformation. And it will also be important to study outlets in languages other than English and with an explicit focus on regional differences. Finally, our findings point to the scope for greater engagement between researchers working on corruption and journalists writing about it, possibly linked to other measures, some of which could be supported by international agencies or non-governmental organisations, such as Transparency International, to provide enhanced training for both researchers and journalists working in this field.

## Conclusions

This research has shown that the Nigerian media is willing to tackle corruption but, by adopting an episodic approach, tends to simplify the issues. The prominence of solutions for corruption in the health sector suggests a public yearning for the issue to be tackled but the solutions proffered focused mainly on less effective measures such enforcement and detection. In depth reporting of a complex phenomenon like corruption is not easy, but we believe that there is scope for improvements through enhanced collaboration between researchers and journalists. But ultimately, change will only occur if, as Mostert and Kaspers note, people are willing to take a stand against corruption. What we found in the Nigerian newspapers is that journalists can play a key role.^39^

## Acknowledgements

The work of DB, EH, and MM are supported by the SOAS Anti-Corruption Evidence (ACE) research consortium funded by UK aid from the UK Government [Contract P0 7073]. The views presented in this publication are those of the authors and do not necessarily reflect the UK government’s official policies or the views of SOAS-ACE or other partner organizations. For more information on SOAS-ACE visit https://ace.soas.ac.uk.

## Ethical issues

The study was approved by the Ethics Committee at London School of Hygiene & Tropical Medicine (LSHTM), London, UK.

## Competing interests

Authors declare that they have no competing interests.

## Authors’ contributions

MAA undertook the review, assisted by MM, and wrote the first draft, under the supervision of MM and DB. MM, DB, and EH then revised the text.

## Supplementary files

Supplementary file 1. PRISMA Flow Diagram for Newspaper Selection and Analysis.

Supplementary file 2. Coding Scheme.
