# Supplementary figures and images for: How Do Nigerian Newspapers Report Corruption in the Health System?
Source: Int J Health Policy Manag. 2020 Mar 14;10(2):77–85. doi: 10.34172/ijhpm.2020.37 (PMC7947671; doi:10.34172/ijhpm.2020.37)

**Supplementary file 1.** PRISMA Flow Diagram for Newspaper Selection and Analysis

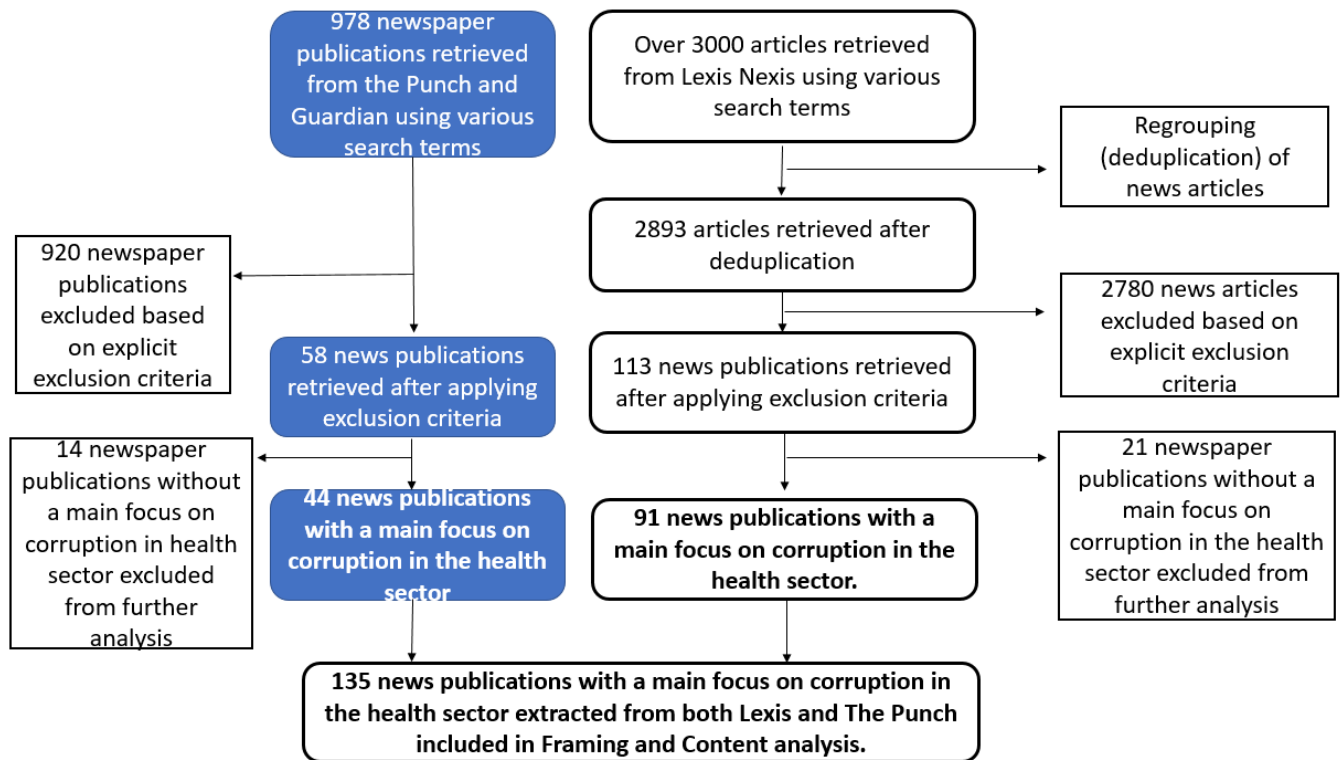

Supplement: Supplementary file 1 — PRISMA Flow Diagram for Newspaper Selection and Analysis. [file ijhpm-10-77-Supp1.pdf]
