# Supplementary material for: How Do Nigerian Newspapers Report Corruption in the Health System?
Source: Int J Health Policy Manag. 2020 Mar 14;10(2):77–85. doi: 10.34172/ijhpm.2020.37 (PMC7947671; doi:10.34172/ijhpm.2020.37)
Supplement: Supplementary file 2 — Coding Scheme. [file ijhpm-10-77-Supp2.pdf]

Supplementary file 2. Coding Scheme

<u>Stage 1</u>. Variables coded were:

a) Tone (Positive, negative, neutral, or ambivalent – see below);

b) Publication source (Newspaper company);

c) Date of publication;

d) Length of Publication (Word Count).

<u>Stage 2</u>. The type of health sector corruption reported in newspaper reports was coded. This was based on the typology and their subtypes (based on Transparency International's typology).

<u>Stage 3</u>. The content analyse used the following 4 "framing functions":

a) The causes of corruption reported as reported by the newspapers;

b) The impacts of corruption in the health sector as reported by the newspapers;

c) Stakeholders held responsible as reported by the newspapers;

d) Solutions proffered as reported by the newspapers.

The phrase "as reported by the media" here is meant to indicate that all sources (News stories, editorials, letters and features) will be considered.

<u>Stage 4</u>

The fourth and final stage of the strategy was a framing analysis. This involved going through the news stories with a view to categorising them as episodic or thematic frames and further analysing each frame. The yardstick for categorising news stories in the episodic frame was if the news article was; a description of specific case of corruption or a narrative based on a stakeholder's perspective (Patient/Public, Provider, payer or regulator) about a specific case of corruption. These frames were then further analysed according to the major issue that emerged.

Note: Tonality is an analysis that uses a subjective assessment to determine if the content of article is either favourable or unfavourable to the person, company, organization or product discussed in the text. Tones were categorized either as positive, negative, neutral, or ambivalent based on how they reported the individual(s) or organization when reporting on a corruption case. Articles which

were neither outrightly positive or negative were categorized as neutral or, where they contained mixed messages, ambivalent. An example of an ambivalent tone is an article reporting on misappropriation of Global Alliance for Vaccine Initiative (GAVI) fund with the headline: “Donor Expresses Disappointment Over Stealing of Healthcare Funds” published in This Day newspaper on 29 July 2016. At first glance, the headline of the article gives an impression of a negative tone, however, the body of the text conveys a positive message as it portrays the government’s willingness and efforts to tackle the issue stating the case is already being investigated. Additionally, the then GAVI chair’s positive remarks of a "breath of fresh air" due to the president’s anticorruption stance and GAVIs willingness to “close the books of the past, and look into future support" gives the article a positive tone. As such this article was categorized as ambivalent.
